# [*N*,*N*,*N*′,*N*′-Tetra­kis(benzimidazol-2-yl­meth­yl)ethane-1,2-diamine]copper(II) sulfate monohydrate

**DOI:** 10.1107/S1600536809043517

**Published:** 2009-10-28

**Authors:** Zuo-an Xiao, Dan Zhan

**Affiliations:** a School of Chemical Engineering and Food Science, Xiangfan University, Xiangfan 441053, People’s Republic of China

## Abstract

In the title compound, [Cu(C_34_H_32_N_10_)]SO_4_·H_2_O, the Cu^II^ ion is coordinated by six N atoms of a hexa­dentate *N*,*N*,*N*′,*N*′-tetra­kis(benzimidazol-2-ylmeth­yl)ethane-1,2-diamine (EDTB) ligand, in a distorted octa­hedral environment. In the crystal structure, inter­molecular N—H⋯O and weak C—H⋯O hydrogen bonds connect the cations, anions and water mol­ecules into a three-dimensional network. The O atoms of the anion are disordered over two sites with refined occupancies of 0.711 (2) and 0.289 (2).

## Related literature

For background information on Cu(II) complexes of benz­imidazole, see: Liao *et al.* (2001 ▶); Qiu *et al.* (2005 ▶). For background to EDTB complexes, see: Chen *et al.* (2004 ▶); Liu *et al.* (2003 ▶); Yang *et al.* (2003 ▶). For the synthesis of EDTB, see: Hendriks *et al.* (1982 ▶). For the treatment of the disordered solvent, see: Spek (2009 ▶). For related structures, see: Athimoolam *et al.* (2005 ▶); Cox *et al.* (2003 ▶); Mohamed *et al.* (2003 ▶); Stähler *et al.* (2001 ▶). 
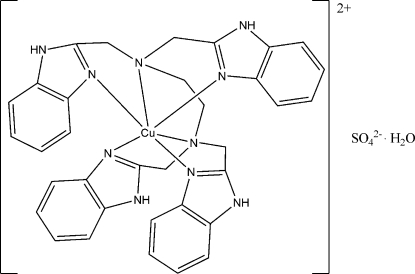


## Experimental

### 

#### Crystal data

[Cu(C_34_H_32_N_10_)]SO_4_·H_2_O
*M* *_r_* = 758.33
Orthorhombic, 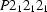

*a* = 11.2955 (8) Å
*b* = 14.4622 (10) Å
*c* = 23.9698 (16) Å
*V* = 3915.7 (5) Å^3^
*Z* = 4
Mo *K*α radiation
μ = 0.66 mm^−1^
*T* = 292 K
0.20 × 0.20 × 0.20 mm

#### Data collection

Bruker SMART CCD area-detector diffractometer
Absorption correction: multi-scan (*SADABS*; Sheldrick, 1996 ▶) *T*_min_ = 0.876, *T*_max_ = 0.876
24117 measured reflections
6894 independent reflections
4146 reflections with *I* > 2σ(*I*)
*R*_int_ = 0.123

#### Refinement

*R*[*F* ^2^ > 2σ(*F* ^2^)] = 0.060
*wR*(*F* ^2^) = 0.124
*S* = 0.86
6894 reflections
504 parameters
8 restraints
H atoms treated by a mixture of independent and constrained refinement
Δρ_max_ = 0.33 e Å^−3^
Δρ_min_ = −0.29 e Å^−3^
Absolute structure: Flack (1983 ▶), 3025 Friedel pairs
Flack parameter: 0.011 (18)

### 

Data collection: *SMART* (Bruker, 2001 ▶); cell refinement: *SAINT-Plus* (Bruker, 2001 ▶); data reduction: *SAINT-Plus*; program(s) used to solve structure: *SHELXS97* (Sheldrick, 2008 ▶); program(s) used to refine structure: *SHELXL97* (Sheldrick, 2008 ▶); molecular graphics: *PLATON* (Spek, 2009 ▶); software used to prepare material for publication: *SHELXTL* (Sheldrick, 2008 ▶).

## Supplementary Material

Crystal structure: contains datablocks global, I. DOI: 10.1107/S1600536809043517/lh2923sup1.cif

Structure factors: contains datablocks I. DOI: 10.1107/S1600536809043517/lh2923Isup2.hkl

Additional supplementary materials:  crystallographic information; 3D view; checkCIF report

## Figures and Tables

**Table 1:** Hydrogen-bond geometry (Å, °)

*D*—H⋯*A*	*D*—H	H⋯*A*	*D*⋯*A*	*D*—H⋯*A*
N4—H4⋯O1^i^	0.86	1.87	2.730 (12)	174
C16—H16⋯O1	0.93	2.59	3.498 (13)	167
N10—H10⋯O3^ii^	0.86	1.88	2.699 (8)	158
N8—H8*A*⋯O4^iii^	0.86	1.91	2.747 (12)	162
N6—H6*A*⋯O1*W*	0.86	1.90	2.743 (10)	167

Symmetry codes: (i) 
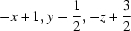
; (ii) 
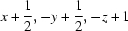
; (iii) 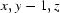.

**Fig. 1.**
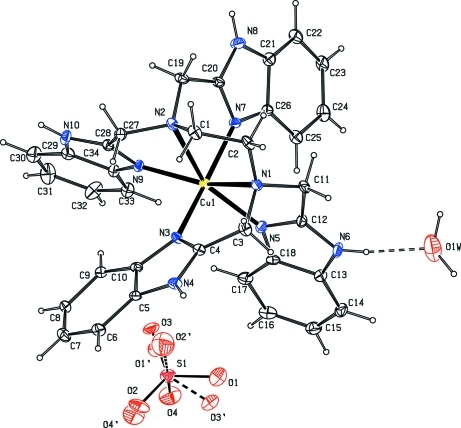
The asymmetric unit of (I), with displacement ellipsoids drawn at the 50% probability level. Thin dashed lines indicate hydrogen bonds and the thick dashed lines indicate the minor component of disorder in the anion.

**Fig. 2.**
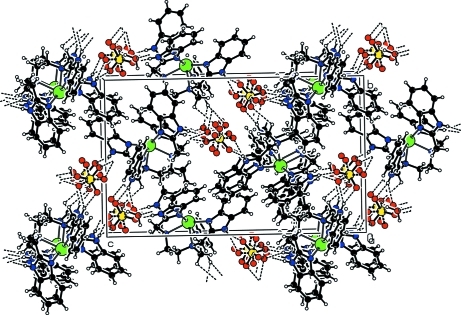
Part of the crystal structure of the title compound with hydrogen bonds shown as dashed lines.

## supplementary crystallographic information

### Comment

Cu(II) complexes of benzimidazole rich ligands have been widely studied in recent years (Liao *et al.*, 2001; Qiu *et al.*, 2005). Several EDTB-metal model compounds have already been reported (Liu *et al.*, 2003; Chen *et al.*, 2004), and the title compound, (I), is part of our effort in this area of research. Herein we report the crystal structure of (I).

In the molecular structure of (I), the Cu^II^ ion is coordinated by four benzimidazole(bzim) N atoms and two amino N atoms of EDTB, forming a distorted octahedral coordination environmemt (Fig.1). Two bzim-N atoms (N3 and N7) occupy the axial positions, the other two bzim-N atoms (N5 and N9) and two amino N atoms(N1 and N2) are located in the equatorial plane. This configuration can be compared with [Cu(EDTB)].2Tos.4H_2_O.C_2_H_5_OH (Tos = 4-methyl-benzene-sulfonate)(Yang *et al.*, 2003). In the crystal structure, intermolecular N—H···O and weak C-H···O hydrogen bonds form a three-dimensional network (Table 1 and Fig.2).

### Experimental

All reagants and slvents were used as obtained without further purification. EDTB was synthesized according to the literature (Hendriks *et al.*,1982). CuSO_4_.5H_2_O (1 mmol, 0.25 g) was dissolved in water (5 mL), and EDTB (1 mmol, 0.58 g) was dissolved in ethanol (40 mL), then the two solutions were mixed and stirred at 333 k for 8 h. The solution was filtered, and the resulting blue precipitate was dissolved in aqueous glycol solution. The green crystals which were formed were obtained after two months. Elemental analysis calculated (with glycol included): C 52.66, H 4.88, N 17.06 %; found: C 52.76, H 4.99, N 16.94 %.

### Refinement

All H atoms bonded to C atoms were placed in calculated positions and constrained to ride on their parent atoms, with C—H distances in the range 0.93–0.97 Å, with *U*_iso_(H) = 1.2*U*_eq_(C). H atoms bonded to N atoms were first found the difference map and then fixed at their ideal positions with N—H = 0.86 Å and *U*_iso_(H) = 1.2*U*_eq_(N). Water H atoms were refined with distance restraints of O—H = 0.85 (1) Å, H···H = 1.39 (1)Å and *U*_iso_(H) = 1.5*U*_eq_(O). The sulfate O atoms O1—O4 are disordered over two positions with the final occupancies being 0.711 (2):0.289 (2) for the major and minor components, respectively. The *DFIX* command (Sheldrick, 2008) was used in the refinement to restrain the S—O bond lengths. During the refinement of the structure, electron-density peaks were located that were believed to be highly disordered solvent molecules (glycol). Attempts made to model the solvent molecules were not successful. The SQUEEZE option in *PLATON* (Spek, 2009) indicated there was a solvent cavity of volume 682.4 Å^3^ containing approximately 121 electrons, *i.e.* four glycol solvent molecules (per cell), which is also corroborated by CHN analysis on a Perkin-Elmer 240C elemental analyzer, i.e. calculated: C 52.66, H 4.88, N 17.06 %; found: C 52.76, H 4.99, N 16.94 %. i.e. every [Cu(EDTB)](SO_4_).H_2_O unit has a glycol solvent molecule, so the whole suitable formula shoud be [Cu(EDTB)(SO_4_).C_2_H_6_O_2_.H_2_O. In the final cycles of refinement, this contribution to the electron density was removed from the observed data. The density, the F(000) value, the molecular weight and the formula are given without taking into account the results obtained with SQUEEZE *PLATON* (Spek, 2009). For a similar treatment of disordered solvent molecules see: Stähler *et al.* (2001); Cox *et al.* (2003); Mohamed *et al.* (2003); Athimoolam *et al.* (2005).

### Crystal data

**Table:**

[Cu(C_34_H_32_N_10_)]SO_4_·H_2_O	*F*(000) = 1572
*M**_r_* = 758.33	*D*_x_ = 1.286 Mg m^−^^3^
Orthorhombic, *P*2_1_2_1_2_1_	Mo *K*α radiation, λ = 0.71073 Å
Hall symbol: P 2ac 2ab	Cell parameters from 2277 reflections
*a* = 11.2955 (8) Å	θ = 2.3–16.6°
*b* = 14.4622 (10) Å	µ = 0.66 mm^−^^1^
*c* = 23.9698 (16) Å	*T* = 292 K
*V* = 3915.7 (5) Å^3^	Block, blue
*Z* = 4	0.20 × 0.20 × 0.20 mm

### Data collection

**Table:**

Bruker SMART CCD area-detector diffractometer	6894 independent reflections
Radiation source: fine-focus sealed tube	4146 reflections with *I* > 2σ(*I*)
graphite	*R*_int_ = 0.123
φ and ω scans	θ_max_ = 25.0°, θ_min_ = 1.6°
Absorption correction: multi-scan (*SADABS*; Sheldrick, 1996)	*h* = −13→13
*T*_min_ = 0.876, *T*_max_ = 0.876	*k* = −16→17
24117 measured reflections	*l* = −28→24

### Refinement

**Table:**

Refinement on *F*^2^	Secondary atom site location: difference Fourier map
Least-squares matrix: full	Hydrogen site location: inferred from neighbouring sites
*R*[*F*^2^ > 2σ(*F*^2^)] = 0.060	H atoms treated by a mixture of independent and constrained refinement
*wR*(*F*^2^) = 0.124	*w* = 1/[σ^2^(*F*_o_^2^) + (0.0454*P*)^2^] where *P* = (*F*_o_^2^ + 2*F*_c_^2^)/3
*S* = 0.86	(Δ/σ)_max_ < 0.001
6894 reflections	Δρ_max_ = 0.33 e Å^−^^3^
504 parameters	Δρ_min_ = −0.28 e Å^−^^3^
8 restraints	Absolute structure: Flack (1983), 3025 Friedel pairs
Primary atom site location: structure-invariant direct methods	Flack parameter: 0.011 (18)

### Special details

**Table:**

Geometry. All e.s.d.'s (except the e.s.d. in the dihedral angle between two l.s. planes) are estimated using the full covariance matrix. The cell e.s.d.'s are taken into account individually in the estimation of e.s.d.'s in distances, angles and torsion angles; correlations between e.s.d.'s in cell parameters are only used when they are defined by crystal symmetry. An approximate (isotropic) treatment of cell e.s.d.'s is used for estimating e.s.d.'s involving l.s. planes.
Refinement. Refinement of *F*^2^ against ALL reflections. The weighted *R*-factor *wR* and goodness of fit *S* are based on *F*^2^, conventional *R*-factors *R* are based on *F*, with *F* set to zero for negative *F*^2^. The threshold expression of *F*^2^ > σ(*F*^2^) is used only for calculating *R*-factors(gt) *etc*. and is not relevant to the choice of reflections for refinement. *R*-factors based on *F*^2^ are statistically about twice as large as those based on *F*, and *R*- factors based on ALL data will be even larger.

### Fractional atomic coordinates and isotropic or equivalent isotropic displacement parameters (Å^2^)

**Table:**

	*x*	*y*	*z*	*U*_iso_*/*U*_eq_	Occ. (<1)
Cu1	0.41029 (6)	0.06897 (4)	0.67483 (3)	0.0402 (2)	
C1	0.5321 (6)	−0.1163 (4)	0.7197 (3)	0.0545 (18)	
H1A	0.6017	−0.0860	0.7348	0.065*	
H1B	0.5482	−0.1821	0.7176	0.065*	
C2	0.4268 (6)	−0.0993 (4)	0.7578 (3)	0.0529 (17)	
H2A	0.3581	−0.1310	0.7429	0.063*	
H2B	0.4435	−0.1250	0.7943	0.063*	
C3	0.4885 (5)	0.0437 (4)	0.7989 (3)	0.0567 (18)	
H3A	0.4483	0.0840	0.8251	0.068*	
H3B	0.5315	−0.0024	0.8201	0.068*	
C4	0.5726 (5)	0.0984 (4)	0.7658 (3)	0.0419 (15)	
C5	0.7267 (6)	0.1826 (4)	0.7440 (3)	0.0432 (16)	
C6	0.8317 (5)	0.2358 (4)	0.7412 (3)	0.0536 (18)	
H6	0.8828	0.2388	0.7716	0.064*	
C7	0.8567 (6)	0.2829 (4)	0.6930 (3)	0.0609 (19)	
H7	0.9246	0.3190	0.6905	0.073*	
C8	0.7806 (6)	0.2762 (4)	0.6483 (3)	0.0519 (17)	
H8	0.7997	0.3082	0.6159	0.062*	
C9	0.6757 (5)	0.2239 (4)	0.6489 (3)	0.0435 (15)	
H9	0.6266	0.2205	0.6178	0.052*	
C10	0.6489 (5)	0.1778 (3)	0.6976 (3)	0.0391 (15)	
C11	0.2773 (6)	0.0178 (4)	0.7808 (3)	0.0525 (18)	
H11A	0.2692	0.0104	0.8208	0.063*	
H11B	0.2229	−0.0244	0.7625	0.063*	
C12	0.2497 (6)	0.1153 (5)	0.7644 (3)	0.0510 (17)	
C13	0.1756 (6)	0.2585 (5)	0.7633 (3)	0.0548 (17)	
C14	0.1185 (6)	0.3424 (5)	0.7727 (3)	0.068 (2)	
H14	0.0694	0.3510	0.8034	0.081*	
C15	0.1372 (6)	0.4103 (5)	0.7355 (4)	0.070 (2)	
H15	0.1017	0.4677	0.7411	0.084*	
C16	0.2068 (6)	0.3970 (4)	0.6900 (3)	0.066 (2)	
H16	0.2152	0.4456	0.6649	0.079*	
C17	0.2669 (6)	0.3141 (4)	0.6788 (3)	0.0576 (17)	
H17	0.3147	0.3067	0.6475	0.069*	
C18	0.2503 (5)	0.2434 (4)	0.7176 (3)	0.0457 (16)	
C19	0.4289 (5)	−0.1352 (4)	0.6300 (3)	0.0528 (17)	
H19A	0.4248	−0.1970	0.6455	0.063*	
H19B	0.4601	−0.1400	0.5924	0.063*	
C20	0.3083 (5)	−0.0949 (4)	0.6279 (2)	0.0441 (16)	
C21	0.1152 (5)	−0.0807 (4)	0.6074 (2)	0.0503 (16)	
C22	0.0023 (6)	−0.0876 (6)	0.5883 (3)	0.067 (2)	
H22	−0.0233	−0.1395	0.5688	0.080*	
C23	−0.0703 (5)	−0.0169 (6)	0.5986 (3)	0.068 (2)	
H23	−0.1482	−0.0210	0.5862	0.082*	
C24	−0.0362 (5)	0.0618 (6)	0.6265 (3)	0.069 (2)	
H24	−0.0910	0.1086	0.6329	0.083*	
C25	0.0780 (5)	0.0718 (4)	0.6451 (2)	0.0514 (15)	
H25	0.1016	0.1248	0.6640	0.062*	
C26	0.1573 (5)	0.0002 (4)	0.6347 (2)	0.0403 (14)	
C27	0.6134 (5)	−0.0561 (4)	0.6309 (2)	0.0482 (16)	
H27A	0.6452	−0.1107	0.6127	0.058*	
H27B	0.6739	−0.0306	0.6552	0.058*	
C28	0.5758 (5)	0.0144 (4)	0.5882 (2)	0.0427 (15)	
C29	0.5699 (5)	0.0998 (4)	0.5127 (3)	0.0465 (16)	
C30	0.5874 (7)	0.1418 (5)	0.4609 (3)	0.0681 (19)	
H30	0.6451	0.1211	0.4362	0.082*	
C31	0.5166 (8)	0.2137 (6)	0.4484 (3)	0.088 (3)	
H31	0.5264	0.2446	0.4147	0.106*	
C32	0.4303 (8)	0.2421 (5)	0.4847 (3)	0.078 (2)	
H32	0.3818	0.2911	0.4743	0.094*	
C33	0.4121 (6)	0.2014 (4)	0.5360 (3)	0.0593 (17)	
H33	0.3538	0.2225	0.5602	0.071*	
C34	0.4840 (5)	0.1282 (4)	0.5497 (2)	0.0419 (15)	
S1	0.2921 (2)	0.62535 (16)	0.56513 (9)	0.0726 (6)	
N1	0.3999 (5)	−0.0027 (3)	0.76355 (19)	0.0453 (12)	
N2	0.5068 (4)	−0.0799 (3)	0.66312 (19)	0.0429 (12)	
N3	0.5531 (4)	0.1224 (3)	0.7127 (2)	0.0397 (12)	
N4	0.6752 (4)	0.1328 (3)	0.7848 (2)	0.0494 (13)	
H4	0.7036	0.1244	0.8177	0.059*	
N5	0.2998 (4)	0.1544 (3)	0.7202 (2)	0.0458 (13)	
N6	0.1771 (5)	0.1751 (4)	0.7923 (2)	0.0619 (15)	
H6A	0.1390	0.1633	0.8225	0.074*	
N7	0.2783 (4)	−0.0116 (3)	0.64646 (19)	0.0421 (12)	
N8	0.2130 (5)	−0.1385 (4)	0.6043 (2)	0.0620 (16)	
H8A	0.2136	−0.1929	0.5899	0.074*	
N9	0.4898 (4)	0.0749 (3)	0.59723 (18)	0.0396 (11)	
N10	0.6270 (4)	0.0264 (4)	0.5393 (2)	0.0547 (15)	
H10	0.6850	−0.0053	0.5261	0.066*	
O1	0.2192 (9)	0.6036 (9)	0.6143 (4)	0.091 (4)	0.711 (16)
O2	0.4106 (11)	0.6476 (11)	0.5839 (8)	0.106 (5)	0.711 (16)
O3	0.2917 (7)	0.5505 (6)	0.5278 (4)	0.083 (4)	0.711 (16)
O4	0.2360 (9)	0.7064 (6)	0.5391 (4)	0.123 (5)	0.711 (16)
O1'	0.2411 (16)	0.6240 (16)	0.5109 (5)	0.080 (9)	0.289 (16)
O3'	0.229 (2)	0.6638 (19)	0.6090 (8)	0.083 (8)	0.289 (16)
O2'	0.337 (3)	0.5324 (12)	0.5775 (16)	0.179 (15)	0.289 (16)
O4'	0.393 (3)	0.688 (3)	0.565 (2)	0.148 (18)	0.289 (16)
O1W	0.0258 (8)	0.1349 (7)	0.8784 (4)	0.179 (4)	
H1WA	0.022 (11)	0.160 (9)	0.918 (5)	0.214*	
H1WB	−0.003 (12)	0.072 (9)	0.871 (6)	0.214*	

### Atomic displacement parameters (Å^2^)

**Table:**

	*U*^11^	*U*^22^	*U*^33^	*U*^12^	*U*^13^	*U*^23^
Cu1	0.0338 (4)	0.0439 (4)	0.0430 (4)	−0.0025 (4)	−0.0032 (4)	−0.0025 (4)
C1	0.051 (4)	0.047 (4)	0.066 (5)	0.003 (3)	−0.007 (4)	0.025 (3)
C2	0.050 (4)	0.053 (4)	0.056 (4)	−0.017 (3)	−0.003 (4)	0.025 (3)
C3	0.045 (4)	0.071 (5)	0.054 (4)	−0.004 (3)	−0.008 (3)	0.012 (4)
C4	0.040 (4)	0.040 (3)	0.046 (4)	0.000 (3)	−0.007 (3)	0.003 (3)
C5	0.052 (4)	0.033 (3)	0.045 (4)	−0.007 (3)	−0.001 (3)	−0.008 (3)
C6	0.030 (4)	0.063 (4)	0.069 (5)	0.002 (3)	−0.018 (3)	−0.011 (4)
C7	0.043 (4)	0.062 (4)	0.078 (6)	−0.009 (3)	−0.004 (4)	0.002 (4)
C8	0.053 (4)	0.045 (4)	0.057 (4)	−0.011 (3)	0.014 (4)	0.000 (3)
C9	0.046 (4)	0.039 (3)	0.046 (4)	−0.001 (3)	0.001 (3)	−0.003 (3)
C10	0.039 (4)	0.030 (3)	0.049 (4)	0.004 (3)	−0.005 (3)	−0.003 (3)
C11	0.060 (5)	0.051 (4)	0.047 (4)	−0.022 (3)	0.012 (4)	0.007 (3)
C12	0.047 (4)	0.067 (5)	0.039 (4)	−0.006 (4)	0.010 (3)	−0.007 (4)
C13	0.055 (4)	0.056 (4)	0.053 (4)	−0.003 (4)	0.004 (4)	−0.011 (4)
C14	0.039 (4)	0.086 (5)	0.078 (6)	0.005 (4)	0.005 (4)	−0.031 (5)
C15	0.069 (5)	0.054 (5)	0.087 (6)	0.004 (4)	−0.006 (4)	−0.015 (5)
C16	0.071 (5)	0.049 (4)	0.078 (6)	0.004 (4)	−0.032 (5)	0.001 (4)
C17	0.069 (5)	0.061 (4)	0.043 (4)	−0.005 (4)	−0.016 (4)	−0.002 (4)
C18	0.048 (4)	0.047 (4)	0.042 (4)	−0.008 (3)	−0.016 (3)	−0.011 (3)
C19	0.037 (4)	0.037 (3)	0.084 (5)	0.001 (3)	−0.009 (4)	−0.007 (3)
C20	0.038 (4)	0.042 (4)	0.052 (4)	−0.007 (3)	−0.009 (3)	−0.006 (3)
C21	0.038 (4)	0.058 (4)	0.055 (4)	0.007 (3)	−0.007 (3)	0.000 (3)
C22	0.036 (4)	0.092 (6)	0.073 (5)	−0.013 (4)	−0.013 (4)	−0.011 (4)
C23	0.022 (4)	0.110 (6)	0.072 (5)	−0.006 (4)	−0.012 (4)	−0.011 (5)
C24	0.036 (4)	0.094 (5)	0.079 (5)	0.011 (4)	−0.006 (4)	0.009 (5)
C25	0.039 (3)	0.051 (3)	0.064 (4)	0.005 (4)	−0.008 (3)	0.009 (3)
C26	0.036 (4)	0.048 (4)	0.037 (3)	−0.003 (3)	−0.005 (3)	−0.002 (3)
C27	0.038 (4)	0.050 (4)	0.057 (4)	0.003 (3)	0.000 (3)	−0.004 (3)
C28	0.034 (4)	0.053 (4)	0.041 (4)	−0.006 (3)	−0.003 (3)	−0.005 (3)
C29	0.038 (4)	0.052 (4)	0.049 (4)	0.001 (3)	−0.013 (3)	0.002 (3)
C30	0.067 (5)	0.098 (5)	0.039 (4)	0.005 (5)	−0.015 (4)	0.002 (4)
C31	0.085 (6)	0.131 (7)	0.048 (5)	0.012 (6)	−0.005 (5)	0.040 (5)
C32	0.100 (7)	0.087 (5)	0.048 (5)	0.040 (5)	−0.014 (5)	0.007 (4)
C33	0.052 (4)	0.075 (5)	0.051 (4)	0.006 (4)	−0.001 (4)	0.001 (4)
C34	0.036 (4)	0.053 (4)	0.036 (4)	0.003 (3)	−0.004 (3)	0.002 (3)
S1	0.0867 (17)	0.0730 (14)	0.0581 (14)	−0.0143 (13)	−0.0084 (13)	−0.0081 (13)
N1	0.040 (3)	0.042 (3)	0.054 (3)	−0.003 (3)	0.005 (3)	0.002 (2)
N2	0.029 (2)	0.049 (3)	0.050 (3)	−0.001 (2)	−0.008 (2)	0.006 (3)
N3	0.030 (3)	0.047 (3)	0.042 (3)	−0.010 (2)	−0.008 (2)	0.005 (2)
N4	0.054 (4)	0.053 (3)	0.041 (3)	−0.007 (3)	−0.017 (3)	−0.002 (3)
N5	0.046 (3)	0.050 (3)	0.042 (3)	0.003 (3)	0.003 (3)	−0.005 (3)
N6	0.053 (4)	0.087 (4)	0.046 (4)	−0.003 (3)	0.021 (3)	−0.014 (3)
N7	0.032 (3)	0.037 (3)	0.057 (3)	0.001 (2)	−0.003 (3)	−0.009 (3)
N8	0.054 (4)	0.053 (3)	0.079 (4)	0.004 (3)	−0.006 (3)	−0.024 (3)
N9	0.032 (3)	0.046 (3)	0.041 (3)	0.006 (3)	0.003 (2)	−0.004 (3)
N10	0.036 (3)	0.082 (4)	0.046 (3)	0.011 (3)	0.005 (3)	−0.011 (3)
O1	0.100 (7)	0.106 (9)	0.065 (6)	0.023 (7)	−0.008 (5)	0.005 (6)
O2	0.094 (8)	0.102 (10)	0.121 (11)	−0.044 (8)	−0.042 (7)	−0.018 (8)
O3	0.072 (6)	0.090 (7)	0.087 (7)	−0.007 (5)	−0.003 (5)	−0.056 (6)
O4	0.174 (11)	0.088 (8)	0.106 (8)	0.010 (7)	−0.012 (7)	0.014 (6)
O1'	0.097 (16)	0.08 (2)	0.059 (13)	−0.002 (13)	−0.010 (11)	0.007 (12)
O3'	0.095 (17)	0.083 (18)	0.070 (16)	0.022 (16)	−0.004 (12)	−0.019 (15)
O2'	0.18 (3)	0.19 (3)	0.17 (4)	−0.01 (2)	0.01 (3)	0.00 (2)
O4'	0.14 (3)	0.15 (4)	0.16 (4)	−0.02 (3)	0.00 (3)	−0.02 (3)
O1W	0.129 (7)	0.246 (11)	0.161 (8)	−0.030 (7)	0.041 (6)	0.028 (9)

### Geometric parameters (Å, °)

**Table:**

Cu1—N3	2.006 (4)	C19—C20	1.482 (8)
Cu1—N7	2.011 (5)	C19—H19A	0.9700
Cu1—N5	2.065 (5)	C19—H19B	0.9700
Cu1—N9	2.067 (4)	C20—N7	1.328 (6)
Cu1—N1	2.369 (4)	C20—N8	1.369 (7)
Cu1—N2	2.429 (5)	C21—C22	1.358 (8)
C1—N2	1.483 (7)	C21—N8	1.387 (7)
C1—C2	1.520 (8)	C21—C26	1.422 (7)
C1—H1A	0.9700	C22—C23	1.334 (9)
C1—H1B	0.9700	C22—H22	0.9300
C2—N1	1.436 (7)	C23—C24	1.376 (9)
C2—H2A	0.9700	C23—H23	0.9300
C2—H2B	0.9700	C24—C25	1.372 (8)
C3—C4	1.469 (7)	C24—H24	0.9300
C3—N1	1.472 (7)	C25—C26	1.392 (7)
C3—H3A	0.9700	C25—H25	0.9300
C3—H3B	0.9700	C26—N7	1.406 (7)
C4—N3	1.337 (7)	C27—N2	1.472 (6)
C4—N4	1.341 (7)	C27—C28	1.506 (7)
C5—N4	1.346 (7)	C27—H27A	0.9700
C5—C6	1.415 (8)	C27—H27B	0.9700
C5—C10	1.419 (8)	C28—N10	1.319 (6)
C6—C7	1.372 (8)	C28—N9	1.324 (7)
C6—H6	0.9300	C29—C34	1.376 (8)
C7—C8	1.376 (8)	C29—N10	1.395 (7)
C7—H7	0.9300	C29—C30	1.396 (8)
C8—C9	1.406 (8)	C30—C31	1.345 (9)
C8—H8	0.9300	C30—H30	0.9300
C9—C10	1.379 (7)	C31—C32	1.369 (9)
C9—H9	0.9300	C31—H31	0.9300
C10—N3	1.394 (7)	C32—C33	1.378 (8)
C11—N1	1.475 (7)	C32—H32	0.9300
C11—C12	1.496 (8)	C33—C34	1.375 (8)
C11—H11A	0.9700	C33—H33	0.9300
C11—H11B	0.9700	C34—N9	1.378 (7)
C12—N5	1.330 (7)	S1—O3'	1.385 (10)
C12—N6	1.366 (7)	S1—O3	1.405 (6)
C13—N6	1.391 (7)	S1—O1'	1.422 (9)
C13—C14	1.393 (8)	S1—O2	1.449 (8)
C13—C18	1.401 (8)	S1—O4'	1.451 (10)
C14—C15	1.343 (9)	S1—O2'	1.467 (10)
C14—H14	0.9300	S1—O4	1.471 (7)
C15—C16	1.359 (9)	S1—O1	1.472 (7)
C15—H15	0.9300	N4—H4	0.8600
C16—C17	1.404 (8)	N6—H6A	0.8600
C16—H16	0.9300	N8—H8A	0.8600
C17—C18	1.394 (8)	N10—H10	0.8600
C17—H17	0.9300	O1W—H1WA	1.02 (12)
C18—N5	1.405 (7)	O1W—H1WB	0.98 (12)
C19—N2	1.430 (6)		
			
N3—Cu1—N7	166.55 (19)	C22—C23—C24	123.5 (6)
N3—Cu1—N5	90.97 (19)	C22—C23—H23	118.2
N7—Cu1—N5	94.41 (19)	C24—C23—H23	118.2
N3—Cu1—N9	92.43 (18)	C25—C24—C23	120.6 (7)
N7—Cu1—N9	92.39 (18)	C25—C24—H24	119.7
N5—Cu1—N9	135.3 (2)	C23—C24—H24	119.7
N3—Cu1—N1	78.56 (18)	C24—C25—C26	118.0 (6)
N7—Cu1—N1	90.77 (18)	C24—C25—H25	121.0
N5—Cu1—N1	76.09 (18)	C26—C25—H25	121.0
N9—Cu1—N1	147.93 (18)	C25—C26—N7	132.9 (5)
N3—Cu1—N2	91.89 (16)	C25—C26—C21	118.6 (5)
N7—Cu1—N2	77.29 (16)	N7—C26—C21	108.5 (5)
N5—Cu1—N2	149.57 (17)	N2—C27—C28	106.5 (4)
N9—Cu1—N2	74.78 (17)	N2—C27—H27A	110.4
N1—Cu1—N2	74.83 (15)	C28—C27—H27A	110.4
N2—C1—C2	109.9 (5)	N2—C27—H27B	110.4
N2—C1—H1A	109.7	C28—C27—H27B	110.4
C2—C1—H1A	109.7	H27A—C27—H27B	108.6
N2—C1—H1B	109.7	N10—C28—N9	112.4 (5)
C2—C1—H1B	109.7	N10—C28—C27	124.7 (5)
H1A—C1—H1B	108.2	N9—C28—C27	122.9 (5)
N1—C2—C1	112.4 (5)	C34—C29—N10	105.0 (5)
N1—C2—H2A	109.1	C34—C29—C30	122.8 (6)
C1—C2—H2A	109.1	N10—C29—C30	132.2 (6)
N1—C2—H2B	109.1	C31—C30—C29	116.8 (7)
C1—C2—H2B	109.1	C31—C30—H30	121.6
H2A—C2—H2B	107.9	C29—C30—H30	121.6
C4—C3—N1	112.0 (5)	C30—C31—C32	120.9 (7)
C4—C3—H3A	109.2	C30—C31—H31	119.5
N1—C3—H3A	109.2	C32—C31—H31	119.5
C4—C3—H3B	109.2	C31—C32—C33	123.0 (7)
N1—C3—H3B	109.2	C31—C32—H32	118.5
H3A—C3—H3B	107.9	C33—C32—H32	118.5
N3—C4—N4	111.6 (5)	C34—C33—C32	117.0 (6)
N3—C4—C3	123.2 (5)	C34—C33—H33	121.5
N4—C4—C3	125.2 (5)	C32—C33—H33	121.5
N4—C5—C6	133.4 (6)	C33—C34—C29	119.5 (6)
N4—C5—C10	105.9 (5)	C33—C34—N9	131.1 (6)
C6—C5—C10	120.6 (6)	C29—C34—N9	109.4 (5)
C7—C6—C5	118.9 (6)	O3'—S1—O3	142.4 (13)
C7—C6—H6	120.6	O3'—S1—O1'	119.5 (15)
C5—C6—H6	120.6	O3'—S1—O2	98.5 (15)
C6—C7—C8	119.5 (6)	O3—S1—O2	111.8 (9)
C6—C7—H7	120.3	O1'—S1—O2	131.4 (11)
C8—C7—H7	120.3	O3—S1—O4'	119 (3)
C7—C8—C9	123.8 (6)	O1'—S1—O4'	109 (2)
C7—C8—H8	118.1	O3'—S1—O2'	113.0 (16)
C9—C8—H8	118.1	O3—S1—O2'	54.8 (15)
C10—C9—C8	117.0 (6)	O1'—S1—O2'	108.2 (17)
C10—C9—H9	121.5	O2—S1—O2'	79.7 (15)
C8—C9—H9	121.5	O4'—S1—O2'	107 (2)
C9—C10—N3	131.9 (5)	O3—S1—O4	110.1 (6)
C9—C10—C5	120.3 (5)	O2'—S1—O4	164.8 (16)
N3—C10—C5	107.8 (5)	O3—S1—O1	110.1 (7)
N1—C11—C12	108.2 (5)	O1'—S1—O1	120.2 (9)
N1—C11—H11A	110.1	O2—S1—O1	108.4 (9)
C12—C11—H11A	110.1	O4'—S1—O1	125 (2)
N1—C11—H11B	110.1	O2'—S1—O1	80.5 (13)
C12—C11—H11B	110.1	O4—S1—O1	105.6 (6)
H11A—C11—H11B	108.4	C2—N1—C3	110.8 (5)
N5—C12—N6	112.0 (6)	C2—N1—C11	114.9 (5)
N5—C12—C11	121.4 (6)	C3—N1—C11	112.7 (5)
N6—C12—C11	126.5 (6)	C2—N1—Cu1	109.2 (3)
N6—C13—C14	132.9 (7)	C3—N1—Cu1	106.5 (3)
N6—C13—C18	104.4 (6)	C11—N1—Cu1	102.1 (3)
C14—C13—C18	122.7 (7)	C19—N2—C27	110.0 (5)
C15—C14—C13	117.2 (7)	C19—N2—C1	115.3 (5)
C15—C14—H14	121.4	C27—N2—C1	113.9 (4)
C13—C14—H14	121.4	C19—N2—Cu1	106.5 (3)
C14—C15—C16	121.4 (7)	C27—N2—Cu1	102.8 (3)
C14—C15—H15	119.3	C1—N2—Cu1	107.2 (3)
C16—C15—H15	119.3	C4—N3—C10	105.6 (5)
C15—C16—C17	123.7 (7)	C4—N3—Cu1	117.6 (4)
C15—C16—H16	118.2	C10—N3—Cu1	136.7 (4)
C17—C16—H16	118.2	C4—N4—C5	109.0 (5)
C18—C17—C16	115.7 (7)	C4—N4—H4	125.5
C18—C17—H17	122.2	C5—N4—H4	125.5
C16—C17—H17	122.2	C12—N5—C18	104.9 (5)
C17—C18—C13	119.3 (6)	C12—N5—Cu1	115.0 (4)
C17—C18—N5	130.3 (6)	C18—N5—Cu1	140.0 (4)
C13—C18—N5	110.3 (6)	C12—N6—C13	108.2 (5)
N2—C19—C20	111.4 (5)	C12—N6—H6A	125.9
N2—C19—H19A	109.4	C13—N6—H6A	125.9
C20—C19—H19A	109.4	C20—N7—C26	106.9 (5)
N2—C19—H19B	109.4	C20—N7—Cu1	116.8 (4)
C20—C19—H19B	109.4	C26—N7—Cu1	135.9 (4)
H19A—C19—H19B	108.0	C20—N8—C21	109.1 (5)
N7—C20—N8	110.8 (5)	C20—N8—H8A	125.4
N7—C20—C19	125.4 (5)	C21—N8—H8A	125.4
N8—C20—C19	123.8 (5)	C28—N9—C34	105.6 (5)
C22—C21—N8	133.2 (7)	C28—N9—Cu1	116.0 (4)
C22—C21—C26	122.0 (6)	C34—N9—Cu1	138.3 (4)
N8—C21—C26	104.7 (5)	C28—N10—C29	107.6 (5)
C23—C22—C21	117.2 (7)	C28—N10—H10	126.2
C23—C22—H22	121.4	C29—N10—H10	126.2
C21—C22—H22	121.4	H1WA—O1W—H1WB	119 (10)
			
N2—C1—C2—N1	−60.5 (7)	N1—Cu1—N2—C27	−135.6 (3)
N1—C3—C4—N3	16.2 (8)	N3—Cu1—N2—C1	62.4 (4)
N1—C3—C4—N4	−166.1 (5)	N7—Cu1—N2—C1	−109.5 (4)
N4—C5—C6—C7	−176.8 (6)	N5—Cu1—N2—C1	−32.7 (5)
C10—C5—C6—C7	0.2 (9)	N9—Cu1—N2—C1	154.4 (4)
C5—C6—C7—C8	−1.0 (9)	N1—Cu1—N2—C1	−15.2 (3)
C6—C7—C8—C9	0.6 (10)	N4—C4—N3—C10	−1.4 (6)
C7—C8—C9—C10	0.5 (9)	C3—C4—N3—C10	176.5 (5)
C8—C9—C10—N3	178.5 (5)	N4—C4—N3—Cu1	175.1 (3)
C8—C9—C10—C5	−1.3 (8)	C3—C4—N3—Cu1	−6.9 (7)
N4—C5—C10—C9	178.7 (5)	C9—C10—N3—C4	−178.3 (6)
C6—C5—C10—C9	1.0 (8)	C5—C10—N3—C4	1.6 (6)
N4—C5—C10—N3	−1.1 (6)	C9—C10—N3—Cu1	6.2 (9)
C6—C5—C10—N3	−178.9 (5)	C5—C10—N3—Cu1	−174.0 (4)
N1—C11—C12—N5	30.3 (8)	N7—Cu1—N3—C4	−40.2 (10)
N1—C11—C12—N6	−147.1 (6)	N5—Cu1—N3—C4	73.4 (4)
N6—C13—C14—C15	179.8 (7)	N9—Cu1—N3—C4	−151.1 (4)
C18—C13—C14—C15	0.2 (10)	N1—Cu1—N3—C4	−2.2 (4)
C13—C14—C15—C16	1.5 (10)	N2—Cu1—N3—C4	−76.3 (4)
C14—C15—C16—C17	−1.9 (11)	N7—Cu1—N3—C10	135.0 (8)
C15—C16—C17—C18	0.6 (9)	N5—Cu1—N3—C10	−111.4 (5)
C16—C17—C18—C13	1.0 (8)	N9—Cu1—N3—C10	24.1 (5)
C16—C17—C18—N5	−177.3 (6)	N1—Cu1—N3—C10	173.0 (5)
N6—C13—C18—C17	178.8 (5)	N2—Cu1—N3—C10	98.9 (5)
C14—C13—C18—C17	−1.5 (9)	N3—C4—N4—C5	0.8 (7)
N6—C13—C18—N5	−2.5 (7)	C3—C4—N4—C5	−177.1 (5)
C14—C13—C18—N5	177.2 (6)	C6—C5—N4—C4	177.6 (6)
N2—C19—C20—N7	9.9 (9)	C10—C5—N4—C4	0.3 (6)
N2—C19—C20—N8	−172.6 (5)	N6—C12—N5—C18	−3.8 (7)
N8—C21—C22—C23	−179.5 (6)	C11—C12—N5—C18	178.4 (5)
C26—C21—C22—C23	−3.4 (10)	N6—C12—N5—Cu1	−179.5 (4)
C21—C22—C23—C24	0.9 (11)	C11—C12—N5—Cu1	2.7 (7)
C22—C23—C24—C25	0.7 (11)	C17—C18—N5—C12	−177.6 (6)
C23—C24—C25—C26	0.1 (9)	C13—C18—N5—C12	3.9 (7)
C24—C25—C26—N7	178.6 (6)	C17—C18—N5—Cu1	−3.6 (10)
C24—C25—C26—C21	−2.4 (8)	C13—C18—N5—Cu1	177.9 (5)
C22—C21—C26—C25	4.2 (9)	N3—Cu1—N5—C12	−98.7 (4)
N8—C21—C26—C25	−178.8 (5)	N7—Cu1—N5—C12	68.9 (4)
C22—C21—C26—N7	−176.6 (6)	N9—Cu1—N5—C12	166.8 (4)
N8—C21—C26—N7	0.5 (6)	N1—Cu1—N5—C12	−20.8 (4)
N2—C27—C28—N10	−151.8 (5)	N2—Cu1—N5—C12	−3.3 (6)
N2—C27—C28—N9	31.6 (7)	N3—Cu1—N5—C18	87.7 (6)
C34—C29—C30—C31	1.0 (10)	N7—Cu1—N5—C18	−104.7 (6)
N10—C29—C30—C31	−178.8 (7)	N9—Cu1—N5—C18	−6.8 (7)
C29—C30—C31—C32	−1.5 (11)	N1—Cu1—N5—C18	165.6 (6)
C30—C31—C32—C33	1.6 (13)	N2—Cu1—N5—C18	−176.9 (5)
C31—C32—C33—C34	−1.1 (11)	N5—C12—N6—C13	2.3 (7)
C32—C33—C34—C29	0.6 (9)	C11—C12—N6—C13	180.0 (6)
C32—C33—C34—N9	178.6 (6)	C14—C13—N6—C12	−179.5 (7)
N10—C29—C34—C33	179.2 (5)	C18—C13—N6—C12	0.2 (7)
C30—C29—C34—C33	−0.6 (9)	N8—C20—N7—C26	0.1 (7)
N10—C29—C34—N9	0.9 (6)	C19—C20—N7—C26	177.9 (5)
C30—C29—C34—N9	−179.0 (5)	N8—C20—N7—Cu1	−173.4 (4)
C1—C2—N1—C3	−73.3 (6)	C19—C20—N7—Cu1	4.4 (8)
C1—C2—N1—C11	157.6 (5)	C25—C26—N7—C20	178.7 (6)
C1—C2—N1—Cu1	43.6 (6)	C21—C26—N7—C20	−0.4 (6)
C4—C3—N1—C2	103.5 (6)	C25—C26—N7—Cu1	−9.6 (10)
C4—C3—N1—C11	−126.3 (5)	C21—C26—N7—Cu1	171.3 (4)
C4—C3—N1—Cu1	−15.1 (6)	N3—Cu1—N7—C20	−47.1 (10)
C12—C11—N1—C2	−158.9 (5)	N5—Cu1—N7—C20	−160.4 (4)
C12—C11—N1—C3	72.9 (6)	N9—Cu1—N7—C20	63.8 (4)
C12—C11—N1—Cu1	−40.9 (5)	N1—Cu1—N7—C20	−84.3 (4)
N3—Cu1—N1—C2	−109.8 (4)	N2—Cu1—N7—C20	−10.0 (4)
N7—Cu1—N1—C2	61.9 (4)	N3—Cu1—N7—C26	141.8 (7)
N5—Cu1—N1—C2	156.3 (4)	N5—Cu1—N7—C26	28.6 (6)
N9—Cu1—N1—C2	−33.8 (6)	N9—Cu1—N7—C26	−107.3 (5)
N2—Cu1—N1—C2	−14.7 (4)	N1—Cu1—N7—C26	104.7 (5)
N3—Cu1—N1—C3	9.8 (4)	N2—Cu1—N7—C26	178.9 (6)
N7—Cu1—N1—C3	−178.4 (4)	N7—C20—N8—C21	0.2 (7)
N5—Cu1—N1—C3	−84.0 (4)	C19—C20—N8—C21	−177.6 (5)
N9—Cu1—N1—C3	85.9 (4)	C22—C21—N8—C20	176.2 (7)
N2—Cu1—N1—C3	105.0 (4)	C26—C21—N8—C20	−0.4 (6)
N3—Cu1—N1—C11	128.2 (4)	N10—C28—N9—C34	1.5 (6)
N7—Cu1—N1—C11	−60.0 (3)	C27—C28—N9—C34	178.5 (5)
N5—Cu1—N1—C11	34.3 (3)	N10—C28—N9—Cu1	−175.9 (4)
N9—Cu1—N1—C11	−155.8 (4)	C27—C28—N9—Cu1	1.0 (7)
N2—Cu1—N1—C11	−136.7 (4)	C33—C34—N9—C28	−179.5 (6)
C20—C19—N2—C27	−126.3 (5)	C29—C34—N9—C28	−1.4 (6)
C20—C19—N2—C1	103.2 (5)	C33—C34—N9—Cu1	−3.0 (10)
C20—C19—N2—Cu1	−15.6 (6)	C29—C34—N9—Cu1	175.1 (4)
C28—C27—N2—C19	72.6 (6)	N3—Cu1—N9—C28	71.9 (4)
C28—C27—N2—C1	−156.1 (4)	N7—Cu1—N9—C28	−95.6 (4)
C28—C27—N2—Cu1	−40.5 (4)	N5—Cu1—N9—C28	165.7 (4)
C2—C1—N2—C19	−76.2 (6)	N1—Cu1—N9—C28	−0.3 (6)
C2—C1—N2—C27	155.2 (5)	N2—Cu1—N9—C28	−19.4 (4)
C2—C1—N2—Cu1	42.2 (5)	N3—Cu1—N9—C34	−104.4 (6)
N3—Cu1—N2—C19	−173.6 (4)	N7—Cu1—N9—C34	88.2 (6)
N7—Cu1—N2—C19	14.4 (4)	N5—Cu1—N9—C34	−10.6 (7)
N5—Cu1—N2—C19	91.2 (5)	N1—Cu1—N9—C34	−176.6 (5)
N9—Cu1—N2—C19	−81.6 (4)	N2—Cu1—N9—C34	164.3 (6)
N1—Cu1—N2—C19	108.8 (4)	N9—C28—N10—C29	−1.0 (6)
N3—Cu1—N2—C27	−57.9 (3)	C27—C28—N10—C29	−177.9 (5)
N7—Cu1—N2—C27	130.1 (3)	C34—C29—N10—C28	0.0 (6)
N5—Cu1—N2—C27	−153.1 (4)	C30—C29—N10—C28	179.9 (6)
N9—Cu1—N2—C27	34.1 (3)		

### Hydrogen-bond geometry (Å, °)

**Table:**

*D*—H···*A*	*D*—H	H···*A*	*D*···*A*	*D*—H···*A*
N4—H4···O1^i^	0.86	1.87	2.730 (12)	174
C16—H16···O1	0.93	2.59	3.498 (13)	167
N10—H10···O3^ii^	0.86	1.88	2.699 (8)	158
N8—H8A···O4^iii^	0.86	1.91	2.747 (12)	162
N6—H6A···O1W	0.86	1.90	2.743 (10)	167

Symmetry codes: (i) −*x*+1, *y*−1/2, −*z*+3/2; (ii) *x*+1/2, −*y*+1/2, −*z*+1; (iii) *x*, *y*−1, *z*.
